# Clinical Characteristics and Outcomes among Vaccinated and Unvaccinated Patients with Cardiovascular Disease Who Were Hospitalized for COVID-19 in Brazil: Retrospective Cohort

**DOI:** 10.3390/vaccines11040861

**Published:** 2023-04-18

**Authors:** Daniele Melo Sardinha, Ana Lúcia da Silva Ferreira, Ricardo José de Paula Souza e Guimarães, Karla Valéria Batista Lima, Luana Nepomuceno Gondim Costa Lima

**Affiliations:** 1 Programa de Pós-Graduação em Biologia Parasitária na Amazônia, Universidade do Estado do Pará and Instituto Evandro Chagas (PPGBPA/UEPA/IEC), Belém 66087-670, Pará, Brazil; 2 Programa de Pós-Graduação em Epidemiologia e Vigilância em Saúde, Instituto Evandro Chagas (PPGEVS/IEC), Ananindeua 67030-000, Pará, Brazil; 3 Laboratório de Geoprocessamento do Instituto Evandro Chagas (LABGEO/IEC), Ananindeua 67030-000, Pará, Brazil; 4 Seção de Bacteriologia e Micologia, Laboratório de Biologia Molecular, Instituto Evandro Chagas (SABMI/LABMOL/IEC), Ananindeua 67030-000, Pará, Brazil

**Keywords:** cardiovascular disease, COVID-19, hospitalized, COVID-19 vaccine, unvaccinated, risk factors

## Abstract

Introduction: COVID-19 in Brazil has already caused, and it still causes, several impacts on health, economy, and education. The risk factors for death involved those with cardiovascular diseases (CVD), which were prioritized for the vaccination of COVID-19. Objective: To investigate the clinical characteristics and outcomes between vaccinated and unvaccinated patients with cardiovascular diseases hospitalized for COVID-19 in Brazil in the year 2022. Methods: A retrospective cohort was analyzed from the year 2022, with cases being hospitalized by COVID-19 being drawn from SIVEP-GRIPE surveillance. We compared clinical characteristics, comorbidities, and outcomes between CVD carriers and non-carriers, and we also compared vaccinated with two doses vs. those that are unvaccinated in CVD carriers. We performed chi-square, odds ratio, logistic regression, and survival analysis. Results: We included, in the cohort, 112,459 hospital inpatients. An amount of 71,661 (63.72%) of the hospitalized patients had CVD. Regarding deaths, 37,888 (33.69%) died. Regarding vaccination against COVID-19, 20,855 (18.54%) people were not vaccinated with any dose among those with CVD. Death *p*- < 0.001 (OR 1.307-CI 1.235–1.383) and fever *p*- < 0.001 (OR 1.156-CI 1.098–1.218) were associated with the unvaccinated CVD carriers, and diarrhea *p*-0.015 (OR 1.116-CI 1.022–1.218), dyspnea *p*-0.022 (OR 1.074-CI 1.011–1.142), and respiratory distress *p*-0.021 (OR 1.070-CI 1.011–1.134) were also recorded. Those patients who possessed predictors of death, including invasive ventilation (*p*- < 0.001 (OR 8.816-CI 8.313–9.350)), were admitted to the ICU *p*- < 0.001 (OR 1.754-CI 1.684–1.827), and some had respiratory distress *p*- < 0.001 (OR 1.367-CI 1.312–1.423), dyspnea *p* < 0.001 (OR 1.341-CI 1.284–1.400), O_2_ saturation < 95% *p*- < 0. 001 (OR 1.307-CI 1.254–1.363), they were unvaccinated against COVID-19 *p*- < 0.001 (OR 1.258-CI 1.200–1.319), they were of male sex *p*- < 0.001 (OR 1.179-CI 1.138–1.221), they had diarrhea *p*-0.018 (OR 1.081-CI 1.013–1.154), and they may have been old *p* < 0.001 (OR 1.034-CI 1.033–1.035). Survival was shorter for the unvaccinated *p*-0.003, and *p*- <0.001. Conclusions: We highlight the predictors of death for those unvaccinated against COVID-19 in this research, and we evidenced the benefits of the COVID-19 vaccine in reducing deaths in hospitalized CVD patients.

## 1. Introduction

COVID-19 in Brazil caused several impacts on health, economy, and education, which was no different than in the rest of the world. However, in Brazil, the policies to combat the disease were affected by the Bolsonaro government at the time, which did not act timely in prevention measures and protocol of care for hospitalized patients. Thus, Brazil faced a health crisis, involving the overcrowding of hospitals, a lack of oxygen supplementation, and sickness of health professionals [1,2,3]. All this caused 698,834 thousand deaths, which were recorded in their highest numbers in 2020 and 2021 [4], which could be minimized by the timely acquisition of vaccines, but the government only started the agreements for the purchase of vaccines in late 2020, and vaccination was started in January 2021 only in health professionals and the elderly over 80 years old, and these treatments were followed slowly. Children were the most affected because the vaccines only started in 2022, and children under one year old only began being vaccinated in early 2023 [5,6,7].

People with comorbidities are already more vulnerable to hospitalization and death by COVID-19; the literature has already reported this in detail [8,9,10]. Thus, they were also groups that suffered the consequences of the lack of public policies to combat the pandemic in Brazil and worldwide [11]. In this sense, when it comes to cardiovascular diseases (CVD), they represent the leading cause of death in Brazil and worldwide, and the main one is acute myocardial infarction (AMI), followed by cerebrovascular events, which are consequences of cardiovascular diseases. In Brazil, about 14 million people have some type of CVD, and, at least 400,000 die each year, representing 30% of all causes of death [12].

The diagnosis and treatment of the main CVDs in Brazil is flawed because the basic health network does not cover all areas, and it does not guarantee the diagnosis, follow-up, and treatment of patients. Systemic arterial hypertension is a silent disease, which is the main risk factor for fatal cardiovascular events, and this is especially true for patients with less education and income, which will be the most affected by fatal events. Other important factors are metabolic syndrome and diabetes mellitus, which also cause cardiovascular events, and the attention network is fragile [13,14,15].

CVD has always been a serious public health problem in Brazil, and we have seen that control programs have not been able to reduce deaths every year. However, with the arrival of the pandemic in the form of COVID-19, which was initially caused by a virus that attacks the respiratory tract, this caused a mild flu-like syndrome or even a severe respiratory condition, requiring hospitalization, oxygen therapy, and intensive care, especially in people with cardiac comorbidities [16,17].

COVID-19 affected the surveillance of all recurrent diseases and illnesses in the country because attention turned to the disease, which became the greatest concern of the population, resulting in deaths at home due to AMI. People were also in fear of seeking hospital services and being infected, and outpatient follow-up in cases of heart failure and other pathologies were interrupted. The consequences were excess deaths in people with CVD [18,19,20].

By rapid, direct, and indirect transmission, as well as without vaccines, COVID-19 rapidly advanced in the country, and it caused dramatic devastation, causing thousands of hospitalizations and deaths [21]. Thus, knowing the clinical profile, comorbidity, and outcome of COVID-19 in hospitalized patients with CVD allows one to evidence clinical and practical conducts in public health for the reduction in hospitalization and death by COVID-19, allowing the development of public policies for reduction, which is based on scientific evidence.

Some studies in the world have already analyzed severe COVID-19 in patients with cardiovascular disease [22,23,24,25]. Additionally, in Brazil, only one cohort study was conducted in the north of the country in the first year of the pandemic, which occurred before vaccination, and this showed what the predictors of death in CVD patients and non-carriers were [26].

In Brazil, no study has analyzed the entire population of severe acute respiratory syndrome (SARS) patients and compared them with patients with CVD, and no studies have evaluated the effect of the vaccine on the clinical pattern and outcome of COVID-19 in pre-existing CVD. Therefore, the aim of this study is to investigate the clinical characteristics and outcomes among vaccinated and unvaccinated patients with cardiovascular disease hospitalized for COVID-19 in Brazil in the year 2022.

## 2. Method

### 2.1. Study Type and Ethical Issues

This is a longitudinal retrospective cohort study based on data from the epidemiological surveillance of acute and severe respiratory syndromes in Brazil, publicly available on the website “(https://opendatasus.saude.gov.br/) (accessed on 20 February 2023)”. These are data from the Influenza Epidemiological Surveillance System platform (SIVEP-GRIPE).

Surveillance of hospitalized patients and deaths from SARS is done through the notification and investigation form of the Influenza Epidemiological Surveillance System (SIVEP-GRIPE), which is filled out by the health professional who starts treating the patient when he/she fits the case definition. The flow of the investigation is to request laboratory tests for respiratory viruses, bacteria, fungi, etc. In Brazil, SIVEP-GRIPE surveillance data are treated and made available on the OpenDataSUS platform of the Brazilian Ministry of Health, which is a platform for data transparency and universal access.

According to Resolution no. 510, OF APRIL 07, 2016, highlights article II, which states that searches that use information of public access, under the terms of Law no. 12.527, of 18 November 2011; III—searches that use information of public domain and V—searches in databases, whose information is aggregated, without the possibility of individual identification, are in effect. They should not be registered or evaluated by the Research Ethics Committee system (CEP/CONEP) [27,28]. Thus, these types of studies are not recommended to be submitted to ethical evaluation and can be freely conducted, since the publicly available data do not contain information, such as name, telephone number, and address of the participant.

All information used in this study was obtained from universally accessible public sources and was therefore not subject to approval by a research ethics committee. All the methods we used to analyze our data were performed in accordance with relevant guidelines and regulations.

This study followed the guidelines of the Report of Observational Studies in Epidemiology (STROBE) [29].

### 2.2. Study Location

It was conducted in Brazil, which is the largest country in South America and in the Latin American region, being the fifth largest in the world in land area (equivalent to 47.3% of the South American territory), with 8,510,345,538 km^2^, and the sixth in population (with more than 207.8 million inhabitants). Brazil’s climate has a wide range of weather conditions over a large area and varied topography, but most of the country is tropical. Brazil’s large land area encompasses different ecosystems, such as the Amazon rainforest, recognized as having the greatest biological diversity in the world. The Amazon rivers provide a variety of habitats, including swamps and streams, each harboring different types of wildlife. The Atlantic forest and the cerrado (savannah) also support great biodiversity, in addition to the caatinga, making Brazil a mega-diverse country. In the South, the Araucaria forest grows under temperate climate conditions [30].

**Figure 1:**
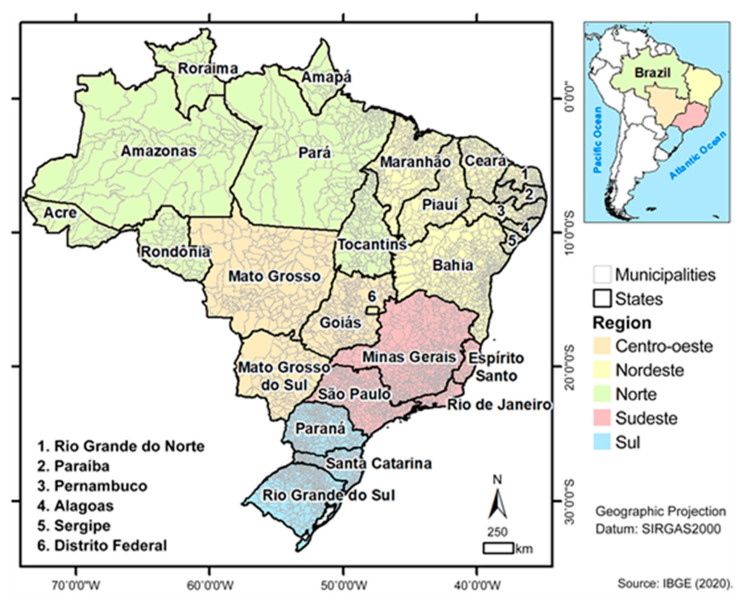
Location of Brazil and its official Brazilian territorial division. Fonte: Sardinha, 2021 [31].

Brazil has five regions, Norte, Nordeste, Centro-oeste, Sodeste, and Sul. They all have geographical, environmental, and population disparities, and the country is characterized by a diversity of races and local vulnerabilities that differ from one another (Figure 1).

### 2.3. Selection of Participants

Cohort time zero was defined as the date of admission, and delta time (∆T) was the period from the date of admission to the outcome (cure or death) for cases with confirmed diagnosis of SARS by COVID-19 real-time polymerase chain reaction (RT-PCR) or antigen. The follow-up time was until the outcome.

The case definition for SARS according to Brazilian surveillance is: an individual with *Syndrome Influenza presenting dyspnea/respiratory distress OR persistent chest pressure OR O2 saturation lower than 95% on room air OR bluish coloration of lips or face. (*SG: Individual with an acute respiratory condition characterized by at least two [2] of the following signs and symptoms: fever (even if referred), chills, sore throat, headache, cough, runny nose, smell or taste disturbances. For the purpose of notification in SIVEP-Gripe, hospitalized cases of SARS or deaths from SARS regardless of hospitalization should be considered [32].

The case definition for cardiovascular diseases was in accordance with the Brazilian Epidemiological Surveillance Guide COVID-19 of 2022, which cites the following diseases: myocardiopathies of various etiologies (heart failure, ischemic myocardiopathy, etc.), hypertension, and cerebrovascular disease [33].

We defined as fully vaccinated those patients with two doses filled in with the date of vaccination and laboratory. For the unvaccinated, we defined patients without any dose registered in the database. We did not consider booster dose because, in Brazil, the laboratory producing the booster dose recommended any immunobiological agent against COVID-19, which deserves an individual analysis because of the risk of bias.

**Figure 2:**
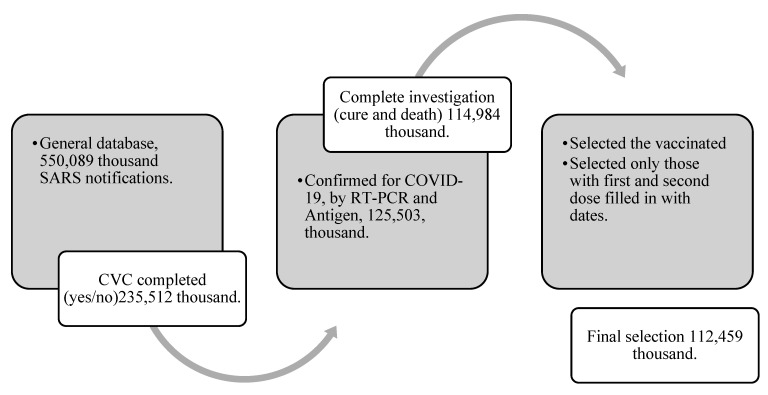
Participant selection flowchart. Source: authors’ research.

The selection of the notifications started by the year of notification, so we used the period from 1 January 2022 to 31 December 2022. We collected the data on 20 February 2023. The details of the selection until the final population are described in the flowchart below (Figure 2).

For the eligibility criteria, we considered cases confirmed for COVID-19 by RT-PCR and antigen criteria, who are residents of Brazil, with field 35 of the investigation form filled in 1 (yes) or 2 (no) with chronic cardiovascular disease, as well as those with the complete outcome, filled in field 80 (cure or death). Cases that did not meet the eligibility criteria were excluded.

After this filter, we still applied the criteria for vaccinated and unvaccinated against COVID-19, and we selected only the cases with the completion of field 35: Did you receive the COVID-19 vaccine? (yes) or (no), and the unfilled fields were excluded. The next filter was the completion of the 1st and 2nd in field 37 and the vaccine laboratory in field 38, and blank cases were excluded. We considered the complete first and second dose schedule, with the vaccine laboratory and dose date filled in.

### 2.4. Data Collection

Data were made available in Excel format, with the variables referring to the notification form of acute and severe respiratory syndromes [32]. The data include demographic, epidemiological, and outcome variables of the investigation, but the database made available for this study does not have the following variables: registration of individuals, name, telephone number, street, house number, neighborhood, zip code, and telephone.

The data were downloaded on 20 February 2023, and the selection of participants was performed, after which the variables were extracted: gender (item 11), age (item 12), state of residence (item 23), signs and symptoms (item 34.), had risk factors/comorbidities (item 35), received COVID-19 vaccine (item 36), date of admission (item 49) was admitted to the ICU (item 53), used ventilatory support (item 56), result of the antigenic test (item 67), result RT-PCR/other method by molecular biology (item 70), final classification (item 78), evolution (item 80), and date of discharge or death (item 81).

The study is subject to information bias due to the use of secondary data from epidemiological surveillance, with the possibility of diagnostic and recording errors and/or impossibility of controlling for possible confounding variables. However, the information bias present is assumed to be of the non-differential type. The form has the variable with chronic cardiovascular disease to mark YES or NO, so it does not specify which cardiovascular disease the individual has.

### 2.5. Data Analysis

The primary endpoint of the study was to evaluate the characteristics of COVID-19 in hospitalized patients with CVD 71,661 (63.72%), and clinical, comorbidity, and outcome characteristics were compared with 40,798 (36.28%) non-carriers. The secondary endpoint was to investigate the clinical characteristics and outcome of COVID-19 in hospitalized CVD patients, vaccinated 59,635 (83.22%) vs. unvaccinated 12,026 (16.78%).

We performed an analysis of the number of notifications in general by state of residence, and we carried out the percentage of CVD carriers, deaths, and those unvaccinated against COVID-19, and we presented these details, descriptively, in a table.

We built two maps with the percentages of CVD and deaths by state of residence of Brazil. We used Microsoft Excel 2019, with the function build graph in the maps model. The software reads by federal unit name and assembles the color map, and, the higher the percentage, the more intense the color. The map also presents a legend on the minimum and maximum in relation to the percentages analyzed by state of the country “(https://support.microsoft.com/pt-br/office/criar-um-gr%C3%A1fico-de-mapas-em-excel-f2cfed55-d622-42cd-8ec9-ec8a358b593b) (accessed on 20 February 2023)”.

Statistical analysis was performed using the program Statistical Package for Social Sciences 20 (SPSS—https://www.ibm.com/analytics/spss-statistics-software, accessed on 20 February 2023), Belém, Pará, Brazil. 

We performed an analysis by age group, comparing CVD carriers with non-carriers, ICU inpatients, unvaccinated against and COVID-19, and death. We present absolute and relative numbers, from tables and graphs, and we applied the adherence test to verify the differences between the proportions of the groups.

In the bivariate analysis, for categorical variables, we used the chi-square statistical tests for the test of independence and Fisher’s exact test (L × C 2 × 2 Contingency Table) for values less than <5. We performed the odds ratio assessment for significant variables (<0.05). In order to identify the associated variables among CVD patients, including clinical, comorbidities, and outcome, we performed the same analysis to compare vaccinated vs. unvaccinated among CVD patients, and we analyzed the clinical and outcome characteristics. 

In the two analyses, in the numerical variables, in this case age, we performed the Kolmogorov-Smirnov normality test to determine whether the test would be parametric or nonparametric, and the test was significant with regards to age in comparing the groups of CVD carriers and non-carriers, as well as vaccinated vs. not vaccinated, and we used the Mann-Whitney test.

We performed two multivariate binary logistic regression models with adjustment for age and sex, the first being with the dependent variable not vaccinated against COVID-19 only in those with CVD, which involved the clinical characteristics, signs, symptoms and outcome, ICU admission, invasive ventilation, and death, to identify the factors associated with unvaccinated hospitalized COVID-19 and CVD patients. The second model was with the dependent variable death, only in those with CVD, to identify predictors of death. Multivariate regression models were performed with the covariates significant in the univariate model < 0.05, and the multivariate model was adjusted by the 2 log likelihood ratio-R2 Nagelkerke-Hosmer and lemeshow tests.

Survival analysis was performed using the outcome of survival (success) and death (failure), considering the date of onset of hospitalization, date of death, and date of discharge from hospital, compared with the dependent variable of not being vaccinated by COVID-19, using the Kaplan-Meier method. We used two tests to check for differences between the vaccinated and unvaccinated groups, the log rank test (Mantel-Cox) and the Breslow test (generalized Wilcoxon).

For all tests, the alpha level of significance was set at 0.05.

## 3. Results

The study analyzed all reported cases of SARS and that were confirmed for COVID-19 in the year 2022 in Brazil, considering the state of residence. After selection of participants, we included in the cohort 112,459 cases that were hospitalized for SARS and confirmed for COVID-19 by laboratory criteria, and 51,694 (45.97%) of these were confirmed by RT-PCR, and 60,765 (54.03%) were confirmed by antigen.

**Figure 3:**
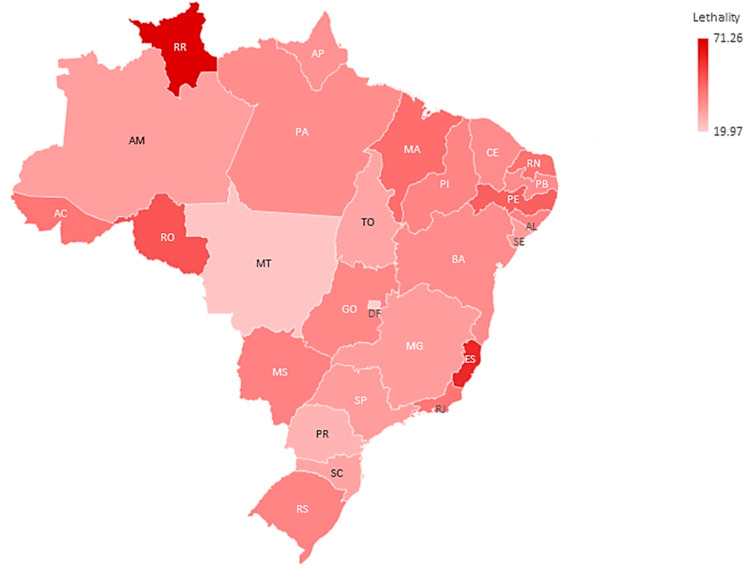
Overall lethality rate per federation unit, in hospitalized patients for COVID-19 in Brazil in 2022. Source: Ministry of Health, Sivep-Gripe/OpenDataSUS, 2022. (software—Microsoft Excel 2019). Microsoft.com. “(accessed on 23 February 2023)”.

**Figure 4:**
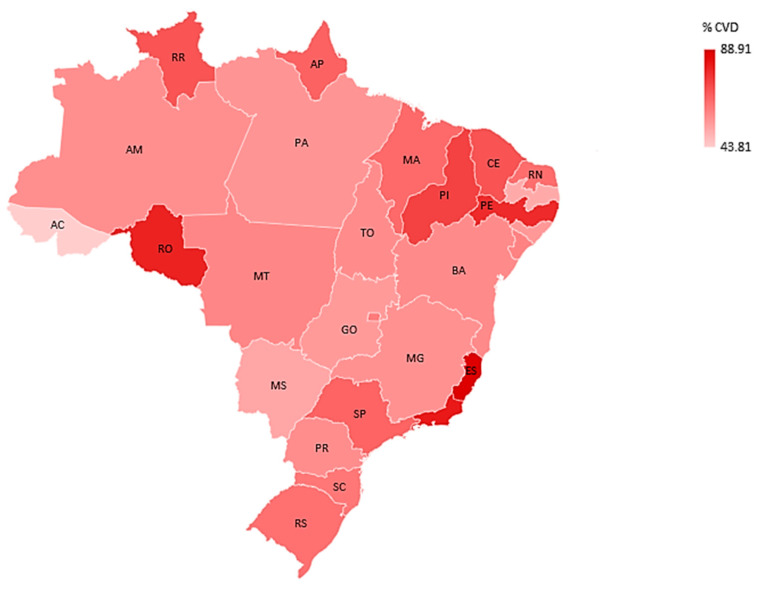
Cardiovascular morbidity rate, per federal unit, in hospitalized patients for COVID-19 in Brazil in 2022. Source: Ministry of Health, Sivep-Gripe/OpenDataSUS, 2022. (software—Microsoft Excel 2019). Microsoft.com “(accessed on 23 February 2023)”.

Among those hospitalized, 71,661 (63.72%) had cardiovascular disease. Regarding deaths, 37,888 (33.69%) died. About the vaccination against COVID-19, 20,855 (18.54%) were not vaccinated with any dose among those with CVD. The states with the highest rates of cardiovascular disease were Espírito Santo (88.91%) and Rio de Janeiro (82.11%). The highest lethality rates were in Roraima (71.26%) and Espírito Santo (60.09%). The highest percentages of unvaccinated were in Tocantins (36.90%) and Alagoas (34.86%) (Table 1, Figure 3 and Figure 4).

In the analysis of clinical characteristics, comparing patients with CVD vs. non-CVD carriers, we identified that being older is associated with CVD carriers *p*-0.061, followed by dyspnea symptom (*p*- < 0.001 (OR 1.119-CI 1.092–1.148)), O_2_ saturation < 95% (*p*- < 0.001 (OR 1.121-CI 1.093–1.149)), other symptoms (*p*-0.001 (OR 1, 044-CI 1.018–1.072)), fatigue (*p*-0.001 (OR 1.044-CI 1.018–1.072)), *p*- < 0.001 (OR 1.818-CI 1.770–1.867), chronic kidney disease (*p*- < 0.001 (OR 1.305 -CI 1.247–1.365)), obesity (*p*- < 0.001 (OR 1.175-CI1.119–1.233)), ICU admission (*p*- < 0.001 (OR 1.198-CI 1.168–1.229)), invasive ventilation (*p*- < 0.001 (OR 1.121-CI 1.084–1.159)), and death (*p*- < 0.001 (OR 1.208-CI 1.177–1.240)) (Table 2).

In Table 3, we analyzed only those with CVD (71,661), and we compared those vaccinated and unvaccinated against COVID-19, to identify the clinical and outcome factors associated with unvaccinated CVD carriers. With regards to age, the youngest were associated with the unvaccinated (*p*- < 0.001), followed by being of male sex (p- 0.002 (OR 1.059-CI 1.119–1.102)), having diarrhea (*p*-0.053 (OR 1.060 = CI 0.988–1.127)), ICU admission (*p*-0.005(OR 1.054-CI 1.013–1.098)), invasive ventilation (*p*- < 0.001(OR 1.167-CI 1.109–1.228)), and death (*p*- < 0. 001(OR 1.156-CI 1.110–1.204)). 

**Figure 5:**
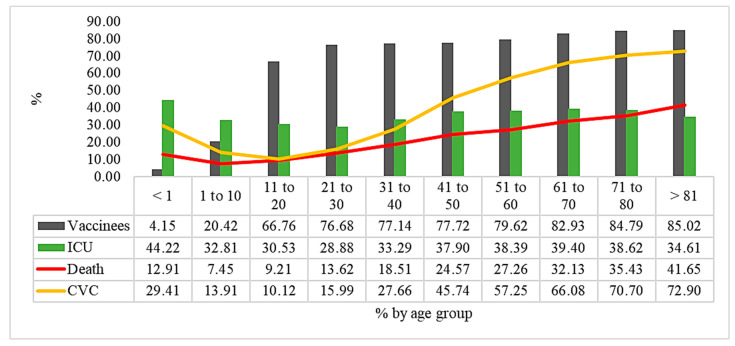
COVID-19 vaccinees, ICU inpatients, CVD patients, and deaths by age group in hospitalized patients with COVID-19 in Brazil in 2022. Source: Ministry of Health, Sivep-Gripe/OpenDataSUS, 2022.

In Figure 5, as well as in Table 4 and Table 5, we show the percentage of vaccinated patients with ICU admission, CVD patients, and deaths. We observed that the age group with the lowest percentage of vaccination against COVID-19 is younger than one year (4.15%), and it was the one that was hospitalized in the ICU (44.42%), with a lethality of (12.91%). Another age group with low vaccination adherence was the 1 to 10 yearsgroup(20.42%), and with ICU hospitalization (32.81%). 

We performed multivariate logistic regression with adjustment for age and sex, and, in the final model, we present the factors associated with unvaccinated hospitalized COVID-19 patients with CVD, and we identified that death is the main factor in unvaccinated COVID-19 cases (*p*- < 0, 001 (OR 1.307-CI 1.235–1.383)), followed by fever (*p*- < 0.001 (OR 1.156-CI 1.098–1.218)), diarrhea (*p*-0.015 (OR 1.116-CI 1.022–1.218)), dyspnea (*p*-0.022 (OR 1.074-CI 1.011–1.142)), and respiratory distress (*p*-0.021 (OR 1.070-CI 1.011–1.134)) (Table 6).

We performed a second multivariate logistic regression model, adjusted for age and sex, to identify the factors associated with deaths in COVID-19 hospitalized CVD patients. We identified that the main factor was invasive ventilation (*p*- < 0.001 (OR 8.816-CI 8.313–9.350)), followed by ICU admissions (*p*- < 0.001 (OR 1.754-CI 1.684–1.827)), respiratory distress (*p*- < 0.001 (OR 1.367-CI 1.312–1.423)), dyspnea (*p* < 0. 001 (OR 1.341-CI 1.284–1.400)), O2 saturation <95% (*p*- < 0.001 (OR 1.307-CI 1.254–1.363)), not being vaccinated against COVID-19 (*p*- < 0.001 (OR 1.258-CI 1.200–1.319)), being of male sex (*p*- < 0.001 (OR 1.179-CI 1.138–1.221)), having diarrhea (*p*-0.018 (OR 1.081-CI 1.013–1.154)) and age (*p* < 0.001 (OR 1.034-CI 1.033–1.035)) (Table 7).

**Figure 6:**
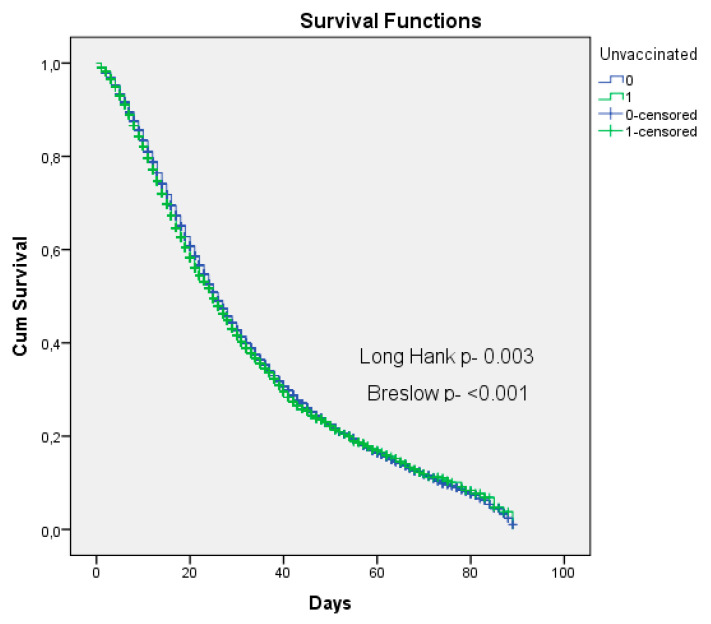
Survival analysis of hospitalized COVID-19 non-vaccinated CVD patients with the outcome death, Brazil, 2022. Source: Ministry of Health, Sivep-Gripe/OpenDataSUS, 2022.

In the survival analysis, we compared two groups with the outcome death, between those vaccinated and those not vaccinated against COVID-19 in hospitalized CVD patients. By the mean number of days of hospitalization until the outcome of cure or death, the unvaccinated patients had a mean of 32 days, and the vaccinated patients’ mean was 33 days. The tests were significant with regards to the log rank (Mantel-Cox)(X2 8.729 *p*-0.003) and the Breslow (Generalized Wilcoxon) test (X2 12.776 *p*- < 0.001) tests, confirming differences in length of stay until outcome between groups. Vaccinated patients have longer survival, and non-vaccinated patients have shorter survival (Figure 6).

## 4. Discussion

This is the first cohort in Brazil to analyze the clinical characteristics, comorbidities, and outcome of COVID-19 in hospitalized patients, with the dependent variable being a carrier of CVD, as well as to verify the factors associated with non-vaccinated patients and the predictors of death in CVD patients.

The overall lethality was 33.69% of those hospitalized by COVID-19, with some disparities among the states of the country. Roraima showed 71.26% deaths. A large cohort study in the first year of the 2020 pandemic in Brazil looked at those hospitalized by COVID-19, and they had a lethality rate of 41.28% higher than our study. However, the lethality by age group showed that the rates in children under 20 years old were very low [34], while in our study, they were higher, especially in <1 year of age, precisely the group with the lowest vaccination rate in Brazil in 2022.

The rate of CVD carriers was 63.72% of reported cases. Another study in 2020 in Brazil, also with hospitalized patients for COVID-19, showed an occurrence of 72.40% of CVD carriers among hospitalized patients [35], a result higher than ours, which may explain the reduction in the vaccine against COVID-19 in the year 2022.

In our study, the case fatality in patients with CVD was 35.21%. A large study of COVID-19 hospital inpatients from 18 countries showed the lethality in CVD patients to be 29.70%, lower than our results. The associations with in-hospital mortality by heart disease subtypes differed considerably, with the strongest association for heart failure (aRR 1.19, 95% CI 1.10–1.30; *p* < 0.018), particularly for severe heart failure (New York Heart Association class III/IV). (aRR 1.41, 95% CI 1.20–1.64; *p* < 0.018). None of the other heart disease subtypes, including ischemic heart disease, remained significant after multivariable adjustment. Severe cardiac complications were diagnosed in <1% of patients [36]. Heart failure was highlighted in one study as a risk factor for mortality in COVID-19, and the authors recommended priority for vaccination [37] similar to a meta-analysis [38]. A limitation of our study was that it was not possible to specify the type of CVD because the notification form has only the variable yes or no for CVD.

Another important point to be highlighted are the possibilities about a better outcome in COVID-19 in patients with CVD, such as the previous use of statins and antihypertensive agents in hospitalized patients, which may be associated with a better outcome [39,40]. However, prospective studies with randomization criteria should be conducted to further clarify this, since, in factors associated with death, CVD still prevails.

When it comes to COVID-19 vaccination to reduce hospitalizations and death, we have already observed, in several studies [41,42,43,44,45,46], including in Brazil, a large study conducted to verify the efficacy of the ChAdOx1 nCoV-19 vaccine (AstraZeneca) in two doses, and the analysis was performed with data from Scotland as well. They showed protection with two doses in a period of up to three months and recommended a booster dose after the third month of the second dose [47].

A large study, also in Brazil, evaluated predictors of death in vaccinated patients, and it observed, in the multivariate analysis, a change in the profile of predictors of death prior to vaccination, in which comorbidities predominated. However, only advanced age > 59 and patients with renal disease remained as predictors of death in this study conducted in 2021 in those hospitalized by COVID-19 [48]. In our analysis, we showed the factors associated with the unvaccinated, which were death, younger age, and clinical signs of SARS severity. However, we limited ourselves only to those with CVD.

Another study in Brazil verified the efficacy of COVID-19 vaccines and showed that the population of Brazil benefited from vaccination in preventing severe outcomes of COVID-19. The results, however, suggest significant reductions in the efficacy of age-specific vaccines given the differences between the age groups 60 to 79 years and over 80 years [49]. We observed, in our cohort, a higher lethality rate in the oldest, even though vaccination rates were higher than the other age groups, which reinforces the importance of the booster dose.

We found the highest lethality rates in the North of the country. Previous research has highlighted inequalities in deaths by geographic region in Brazil, as well as that the associated factors are directly related to local vulnerabilities and quality of health care [50].

When it comes to local vulnerabilities, we found only one cohort in Brazil, which compared the clinical characteristics and outcome of COVID-19 between CVD carriers and non-carriers in the first year of the pandemic, before vaccination in a northern state in the Amazon region. Additionally, we highlighted the predictors of death associated with CVD patients, which were similar to non-carriers. However, the odds ratio in CVD was higher, as well as the significance in deaths < 59 years in CVD patients and cough as an independent factor, which still showed a lethality 55.5% higher than other studies in the literature and our results [26]. We know that this region is already vulnerable due to local factors, including environmental factors, indigenous peoples, and environmental contamination by mercury [51,52,53].

Relative to other countries, a cohort in Italy bought the clinical and laboratory characteristics of CVD and non-carriers hospitalized for COVID-19 and identified that patients hospitalized with concomitant heart disease and COVID-19 have an extremely poor prognosis compared to individuals without a history of heart disease, with higher mortality, thromboembolic events, and septic shock rates [54]. Another cohort in Iran also made the same comparison and showed the same in 660 hospitalized patients with CVD and COVID-19. They showed a significant correlation between the mortality rate of cardiovascular patients with COVID-19 and symptoms, such as headache, loss of consciousness, oxygen saturation less than 93%, and the need for mechanical ventilation [55].

A meta-analysis showed that cardiovascular disease was associated with increased poor composite outcome (RR 2.23 [1.71,2.91], *p* < 0.001; I 2: 60%), mortality (RR 2.25 [1.53,3.29], *p* < 0.001; I 2: 33%) and severe COVID-19 (RR 2.25 [1.51,3.36], *p* < 0.001; I 2: 76%). Meta-regression demonstrated that the association was not influenced by gender, age, hypertension, diabetes, and respiratory comorbidities. Furthermore, the association between cerebrovascular disease and poor outcome was not affected by cardiovascular disease and vice versa [56]. Another meta-analysis also highlighted the higher risk of acute cardiovascular complications in preexisting CVD patients hospitalized for COVID-19, such as acute cardiac injury, which contributes to the outcome death [57].

In our cohort of CVD patients, we showed that the unvaccinated have a higher chance of death, considering both doses of the COVID-19 vaccines. One study evaluated both doses of the vaccines. In the fully vaccinated, there was 12.5% (23/184) mortality, while it was 31.45% (309/984) among the unvaccinated (OR 0.3, 95% CI 0.2 to 0.5, *p* < 0.0001). In the multivariate model, complete vaccination status and younger age were associated with survival [58].

We did not locate any studies in the literature similar to our cohort, as our research was limited to CVD, to verify the factors associated with unvaccinated CVD patients. The other studies compare all groups of hospitalized patients. However, when it comes to the change in profile of the vaccinated hospitalized patients, studies have observed the change in pattern, as well as in young men with no comorbidities that were not vaccinated [59,60,61]. A study in Brazil in 2021 analyzed the impacts of vaccination on deaths in the elderly by COVID-19 and showed a decrease in deaths in elderly >80 years because, at the time, they were priority groups, and the other age groups had not yet been vaccinated, and deaths remained [62]. We showed the highest rate of CVD in elderly >81 years. Thus, it is a group at high risk for age and presence of comorbidities, and we saw that vaccination can minimize deaths.

A cohort in Israel showed that the deaths that still occur even in fully vaccinated individuals are mainly associated with the presence of CVD in conjunction with other comorbidities, which increase the severity and death. They also recommend that this group of multiple comorbidities should be vaccinated and continue to adopt all preventive measures, such as the use of masks, social distancing, and hand hygiene, i.e., pharmacological and non-pharmacological measures should be used in this group [63].

Studies have already analyzed cardiovascular events after the use of the vaccines against COVID-19 and showed that there was no increase in severe cardiovascular events in patients with CVD vaccinated against COVID-19, such as ischemia or hemorrhage, as well as no difference in comparison with the control group, showing safety of the vaccine in patients with CVD [64,65]. 

Regarding SARS in people with CVD, a study has also shown safety and reduction of cardiovascular events in influenza vaccinees, as well as reduction in all-cause deaths [66]. In the future, we expect to see similar benefits from the influenza vaccine.

Vaccinating people with CVD is a health policy to prevent and reduce deaths, reduce spending on hospital admissions, including the ICU, which has a high cost, because CVD is already an old public health problem in the country. It is worth mentioning the importance of the influenza vaccine, as well, so Brazil should prioritize vaccinating this population because it will cost less in the long run and will provide quality of life and health to Brazilians [67].

We highlight, in this study, ICU admissions in the age group under one year, and this was the age group that accounted for the most (44%). It is a reflection of the delay in policies and authorization of the COVID-19 vaccination in the under one-year-olds. At the moment, we realize that this is a group that should be prioritized urgently for vaccination throughout Brazil. By January 2023, vaccination had not been started in this public in the country.

The limitation of our cohort is based on the risk of bias because it is secondary data from epidemiological surveillance, as well as the completion of the variable carrier or not of CVD, which may be even more present in COVID-19 in hospitalized patients, but there is the risk of not completing the variable, as well as that the blanks were excluded from the analysis. Vaccination data are reliable because the Brazilian Ministry of Health automatically transfers vaccination data from the immunization system to the other epidemiological surveillance systems. Therefore, those who are not vaccinated have not been reported in the immunization system, and therefore they have not been vaccinated.

It is also worth noting, as a limitation of the study, death by clinical judgment, because all selected cases were hospitalized for SARS, which is the mandatory case definition for the purpose of notification in SIVEP-GRIPE in Brazil. Therefore, all were also laboratory confirmed for COVID-19, but death by clinical judgment is a risk of bias in epidemiological studies.

## 5. Conclusions

We showed that the factors associated with death in those hospitalized for COVID-19 and with CVD are still similar before the vaccine, such as invasive ventilation, admitted to ICU, respiratory distress, dyspnea, O_2_ saturation < 95%, being of male gender, and having diarrhea. However, the variable “Not vaccinated against COVID-19” stood out with an odds ratio for death in patients with CVD.

We also evidenced the factors associated with non-vaccinated CVD carriers, the first being death, followed by fever, diarrhea, dyspnea, and respiratory distress. This clarifies the importance of vaccination against COVID-19 in this vulnerable group as a protective factor against death.

Thus, CVD remains a very important risk factor for death in COVID-19, but we have seen that vaccination against the disease minimizes deaths, and non-pharmacological measures should also be adopted for the prevention of COVID-19 in this risk group, since CVD is most often not an isolated comorbidity in the patient.

Public policies should be directed to increase vaccination coverage in this public, as well as to highlight the importance of the booster dose, as it will reflect in fewer hospitalizations, deaths, and costs to the health system. It is also important to strengthen the program for hypertension and other CVDs in primary health care, as well as for the early diagnosis and treatment of cardiovascular diseases in this population, especially the most vulnerable in education and income.

## Figures and Tables

**Table 1:** CVD carriers, deaths, and those unvaccinated against COVID-19, per federation unit, in hospitalized patients for COVID-19 in Brazil in 2022.

State of Residence	CVC	%	Death	%	Unvaccinated	%	Total	%
AC	85	43.81	81	41.75	55	28.35	194	0.17
AL	484	55.5	338	38.76	304	34.86	872	0.78
AM	599	57.43	331	31.74	325	31.16	1043	0.93
AP	127	67.2	66	34.92	43	22.75	189	0.17
BA	2395	58.6	1483	36.29	961	23.51	4087	3.63
CE	2257	70.42	1166	36.38	866	27.02	3205	2.85
DF	1643	61.91	530	19.97	506	19.07	2654	2.36
ES	401	88.91	271	60.09	151	33.48	451	0.4
GO	1928	55.16	1315	37.63	844	24.15	3495	3.11
MA	392	65.77	262	43.96	171	28.69	596	0.53
MG	9503	57.14	5320	31.99	2728	16.4	16,632	14.79
MS	1162	52.58	864	39.1	577	26.11	2210	1.97
MT	609	59.53	228	22.29	201	19.65	1023	0.91
PA	852	56.42	545	36.09	440	29.14	1510	1.34
PB	1037	51.62	737	36.68	395	19.66	2009	1.79
PE	767	78.67	459	47.08	236	24.21	975	0.87
PI	624	74.2	323	38.41	148	17.6	841	0.75
PR	5621	57.95	2580	26.6	1585	16.34	9699	8.62
RJ	5705	82.11	2913	41.93	1273	18.32	6948	6.18
RN	698	64.75	463	42.95	210	19.48	1078	0.96
RO	235	80.2	146	49.83	64	21.84	293	0.26
RR	61	70.11	62	71.26	28	32.18	87	0.08
RS	6132	64.1	3676	38.42	1402	14.65	9567	8.51
SC	3314	62.74	1610	30.48	1081	20.47	5282	4.7
SE	577	60.23	310	32.36	239	24.95	958	0.85
SP	24224	66.98	11690	32.32	5877	16.25	36,168	32.16
TO	229	58.27	119	30.28	145	36.90	393	0.35
BRAZIL	71661	63.72	37888	33.69	20,855	18.54	112,459	100

Source: Ministry of Health, Sivep-Gripe/OpenDataSUS, 2022.

**Table 2:** Clinical characteristics, comorbidities, and outcomes of COVID-19 in hospitalized patients with CVD vs. non-carriers of CVD in Brazil in 2022.

Characteristics	Total (112,459)	%	CVD Carriers (71,661)	%	Non-CVD Carriers (40,798)	%	*p*-Value	OR	IC 95%	
Age							<0.001 *			
Minim	0		0		0					
Median	74		62		45					
Maxim	118		118		114					
Male	55,402	49.26	35,178	49.09	20,224	49.57	0.061			
Fever	48,825	43.42	29,584	41.28	19,241	47.16	<0.001	0.778	0.769	0.807
Cough	72,705	64.65	46,376	64.72	26,329	64.54	0.273			
Sore throat	17,202	15.3	10,759	15.01	6443	15.79	<0.001	0.942	0.911	0.974
Dyspnoea	70,628	62.8	45,692	63.76	24,936	61.12	<0.001	1.119	1.092	1.148
Respiratory distress	56,518	50.26	35,740	49.87	20,778	50.93	<0.001	0.959	0.936	0.982
O_2_ saturation < 95%	67,380	59.92	43,649	60.91	23,731	58.17	<0.001	1.121	1.093	1.149
Diarrhoea	9547	8.49	5871	8.19	3676	9.01	<0.001	0.901	0.863	0.941
Vomit	9182	8.16	5480	7.65	3702	9.07	<0.001	0.830	0.794	0.867
Another symptom	36,502	32.46	23,507	32.8	12,995	31.85	0.001	1.044	1.018	1.072
Abdominal pain	6898	6.13	4070	5.68	2828	6.93	<0.001	0.808	0.769	0.850
Fatigue	26,282	23.37	16,876	23.55	9406	23.06	0.030	1.028	0.999	1.058
Loss of smell	2710	2.41	1600	2.23	1110	2.72	<0.001	0.817	0.756	0.882
Loss of taste	2916	2.59	1756	2.45	1160	2.84	<0.001	0.858	0.796	0.925
Chronic hematological disease	1953	1.74	878	1.23	1075	2.63	<0.001	0.458	0.419	0.512
Down Syndrome	643	0.57	307	0.43	336	0.82	<0.001	0.518	0.444	0.605
Chronic liver disease	1825	1.62	977	1.36	848	2.08	<0.001	0.651	0.593	0.714
Asthma	4332	3.85	2097	2.93	2235	5.48	<0.001	0.520	0.489	0.553
Diabetes Mellitus	38,605	34.33	27,976	39.04	10,629	26.05	<0.001	1.818	1.770	1.867
Chronic neurological disease	12,986	11.55	7269	10.14	5717	14.01	<0.001	0.693	0.668	0.719
Another chronic pneumopathy	10,291	9.15	6543	9.13	3748	9.19	0.381			
Immunodepression/immunosuppression	7018	6.24	2823	3.94	4195	10.28	<0.001	0.358	0.341	0.376
Chronic kidney disease	9521	8.47	6585	9.19	2936	7.2	<0.001	1.305	1.247	1.365
Obesity	7945	7.06	5332	7.44	2613	6.4	<0.001	1.175	1.119	1.233
ICU admission	41,474	36.88	27,512	38.39	13,962	34.22	<0.001	1.198	1.168	1.229
Invasive ventilation	18,085	16.08	11,919	16.63	6166	15.11	<0.001	1.121	1.084	1.159
Death	37,888	33.69	25,229	35.21	12,659	31.03	<0.001	1.208	1.177	1.240
Unvaccinated against COVID-19	20,855	18.54	12,026	16.78	8829	21.64	<0.001	0.730	0.708	0.753

Source: Ministry of Health, Sivep-Gripe/OpenDataSUS, 2022. Chi-squared test, * Mann-Whitney test, OR (odds ratio), CI (confidence interval).

**Table 3:** Clinical and outcome factors of COVID-19 in hospitalized CVD patients associated with those unvaccinated against COVID-19 in Brazil in 2022.

Characteristics	Total (71,661)	%	Vaccinated (59,635)	%	Unvaccinated (12,026)	%	*p*-Value	OR	IC 95%	
Age							<0.001 *			
Minimum	0		0		0					
Median	76		77		74					
Maximum	118		118		108					
Male	35,178	49.09	29,130	48.85	6048	50.29	0.002	1.059	1.119	1.102
Fever	29,584	41.28	24,365	40.86	5219	43.4	<0.001	1.110	1.067	1.155
Cough	46,376	64.72	38,829	65.11	7547	62.76	<0.001	0.903	0.867	0.940
Sore throat	10,759	15.01	9042	15.16	1717	14.28	0.007	0.932	0.881	0.985
Dyspnea	45,692	63.76	37,959	63.65	7733	64.3	0.090			
Respiratory distress	35,740	49.87	29,667	49.75	6073	50.5	0.068			
O_2_ saturation < 95%	43,649	60.91	36,300	60.87	7349	61.11	0.316			
Diarrhea	5871	8.19	4841	8.12	1030	8.56	0.053	1.060	0.988	1.127
Vomit	5480	7.65	4588	7.69	892	7.42	0.154			
Abdominal pain	4070	5.68	3463	5.81	607	5.05	0.138			
Fatigue	16,876	23.55	14,322	24.02	2554	21.24	0.035	0.953	0.906	1.003
Loss of smell	1600	2.23	1344	2.25	256	2.13	0.298			
Loss of taste	1756	2.45	1467	2.46	289	2.4	0.139			
Another symptom	23,507	32.8	19,552	32.79	3955	32.89	0.419			
ICU admission	27,512	38.39	22,769	38.18	4743	39.44	0.005	1.054	1.013	1.098
Invasive ventilation	11,919	16.63	9697	16.26	2222	18.48	<0.001	1.167	1.109	1.228
Death	25,229	35.21	20,660	34.64	4569	37.99	<0.001	1.156	1.110	1.204

Source: Ministry of Health, Sivep-Gripe/OpenDataSUS, 2022. Chi-squared test, * Mann-Whitney test, OR (odds ratio), CI (confidence interval).

**Table 4:** Cardiovascular disease and ICU admissions by age group in hospitalized COVID-19 patients in Brazil in 2022.

	Cardiovascular Disease				Total		*p*-Value
**Age Group**	No	%	Yes	%	*n*	%	<0.001
<1	629	70.59	262	29.41	891	0.79	
1 a 10	1467	86.09	237	13.91	1704	1.52	
11 a 20	995	89.88	112	10.12	1107	0.98	
21 a 30	1949	84.01	371	15.99	2320	2.06	
31 a 40	2560	72.34	979	27.66	3539	3.15	
41 a 50	3118	54.26	2628	45.74	5746	5.11	
51 a 60	4740	42.75	6348	57.25	11,088	9.86	
61 a 70	6935	33.92	13,513	66.08	20,448	18.18	
71 a 80	8284	29.30	19,991	70.70	28,275	25.14	
>81	10,121	27.10	27,220	72.90	37,341	33.20	
**Total**	40,798	36.28	71,661	63.72	112,459	100.00	
	ICU Admitted						
**Age Group**	No	%	Yes	%	*n*	%	<0.001
<1	497	55.78	394	44.22	891	0.79	
1 a 10	1145	67.19	559	32.81	1704	1.52	
11 a 20	769	69.47	338	30.53	1107	0.98	
21 a 30	1650	71.12	670	28.88	2320	2.06	
31 a 40	2361	66.71	1178	33.29	3539	3.15	
41 a 50	3568	62.10	2178	37.90	5746	5.11	
51 a 60	6831	61.61	4257	38.39	11,088	9.86	
61 a 70	12,392	60.60	8056	39.40	20,448	18.18	
71 a 80	17,355	61.38	10,920	38.62	28,275	25.14	
>81	24,417	65.39	12,924	34.61	37,341	33.20	
**Total**	70,985	63.12	41,474	36.88	112,459	100.00	

Source: Ministry of Health, Sivep-Gripe/OpenDataSUS, 2022. Chi-square test.

**Table 5:** Deaths and unvaccinated against COVID-19 by age group in hospitalized patients with COVID-19 in Brazil in 2022.

	Death						
Age Group	No	%	Yes	%	*n*	%	<0.001
<1	776	87.09	115	12.91	891	0.79	
1 a 10	1577	92.55	127	7.45	1704	1.52	
11 a 20	1005	90.79	102	9.21	1107	0.98	
21 a 30	2004	86.38	316	13.62	2320	2.06	
31 a 40	2884	81.49	655	18.51	3539	3.15	
41 a 50	4334	75.43	1412	24.57	5746	5.11	
51 a 60	8065	72.74	3023	27.26	11,088	9.86	
61 a 70	13,878	67.87	6570	32.13	20,448	18.18	
71 a 80	18,258	64.57	10,017	35.43	28,275	25.14	
>81	21,790	58.35	15,551	41.65	37,341	33.2	
Total	74,571	66.31	37,888	33.69	112,459	100	
	Vaccine from COVID-19						
Age Group	No	%	Yes	%	*n*	%	<0.001
<1	854	95.85	37	4.15	891	0.79	
1 a 10	1356	79.58	348	20.42	1704	1.52	
11 a 20	368	33.24	739	66.76	1107	0.98	
21 a 30	541	23.32	1779	76.68	2320	2.06	
31 a 40	809	22.86	2730	77.14	3539	3.15	
41 a 50	1280	22.28	4466	77.72	5746	5.11	
51 a 60	2260	20.38	8828	79.62	11,088	9.86	
61 a 70	3490	17.07	16,958	82.93	20,448	18.18	
71 a 80	4302	15.21	23,973	84.79	28,275	25.14	
>81	5595	14.98	31,746	85.02	37,341	33.2	
Total	20,855	18.54	91,604	81.46	112,459	100	

Source: Ministry of Health, Sivep-Gripe/OpenDataSUS, 2022. Chi-square test.

**Table 6:** Final multivariate model for the factors associated with the unvaccinated against COVID-19 in hospitalized CVD patients in Brazil in 2022.

Variables	*p*-Value	OR *	** CI 95%	
Death	<0.001	1.307	1.235	1.383
Fever	<0.001	1.156	1.098	1.218
Diarrhea	0.015	1.116	1.022	1.218
Dyspnea	0.022	1.074	1.011	1.142
Respiratory distress	0.021	1.070	1.011	1.134
Age	<0.001	0.979	0.978	0.980
Admitted to ICU	0.016	0.935	0.885	0.988
Cough	0.014	0.933	0.883	0.986
Sore throat	0.036	0.928	0.864	0.995
Fatigue	0.009	0.925	0.872	0.980
Vomit	0.038	0.904	0.821	0.994
Abdominal pain	0.053	0.903	0.814	1.001
Constant	0.000	0.741		

Source: Ministry of Health, Sivep-Gripe/OpenDataSUS, 2022. Multivariate (2 log likelihood 40719,117a—*p*-value < 0.001), (R2 Nagelkerke = 0.030), (Hosmer and lemeshouw test—*p*-value 0.001). * Confidence Interval (95%). ** Odds Ratio.

**Table 7:** Final multivariate model for factors associated with deaths in hospitalized CVD patients in Brazil in 2022.

Variables	*p*-Value	* OR	** CI 95%	
Invasive ventilation	<0.001	8.816	8.313	9.350
Admitted to ICU	<0.001	1.754	1.684	1.827
Respiratory distress	<0.001	1.367	1.312	1.423
Dyspnea	<0.001	1.341	1.284	1.400
O_2_ saturation < 95%	<0.001	1.307	1.254	1.363
Unvaccinated against COVID-19	<0.001	1.258	1.200	1.319
Male	<0.001	1.179	1.138	1.221
Diarrhea	0.018	1.081	1.013	1.154
Age	<0.001	1.034	1.033	1.035
Another symptom	<0.001	0.869	0.836	0.903
Sore throat	<0.001	0.721	0.683	0.760
Cough	<0.001	0.682	0.657	0.708
Constant	<0.001	0.018		

Source: Ministry of Health, Sivep-Gripe/OpenDataSUS, 2022. Multivariate (2 log likelihood 74390,014a—*p*-value < 0.001), (R2 Nagelkerke = 0.314), (Hosmer and lemeshouw test—*p*-value 0.001). * Confidence Interval (95%). ** Odds Ratio.

## Data Availability

The data are public with universal access, available on the Ministry of Health’s transparency platform from Brazil https://opendatasus.saude.gov.br/, “(accessed on 23 February 2023)”.
